# Exploring a Ugi‐Mediated Polymerization‐Induced Self‐Assembly Approach to Peptoid Particles

**DOI:** 10.1002/marc.70389

**Published:** 2026-08-28

**Authors:** Thi Phuong Thu Nguyen, Maria Cristina Molaro, Hao‐Ning Tien, Lei Lei, Pierre Stiernet, Clemence Le Coeur, Federica Sodano, Antoine Debuigne, Benoit Couturaud

**Affiliations:** ^1^ Univ Paris Est Creteil, CNRS, Institut de Chimie et des Matériaux Paris‐Est(ICMPE) UMR 7182 Thiais France; ^2^ Laboratoire De Chimie de La Matière Condensée De Paris UMR 7574 Sorbonne Université Paris France; ^3^ Department of Pharmacy School of Medicine University of Naples Federico II Naples Italy; ^4^ School of Automotive Materials Hubei University of Automotive Technology Shiyan People's Republic of China; ^5^ Center for Education and Research on Macromolecules (CERM) CESAM Research Unit University of Liege (ULiege) Liège Belgium; ^6^ Laboratoire Léon Brillouin CEA‐CNRS (UMR‐12) CEA Saclay Université Paris‐Saclay Gif‐sur‐Yvette France

**Keywords:** nanoparticle, polycondensation, polymerization induced self‐assembly, synthetic methods, ugi

## Abstract

This work investigates the synthesis of polypeptoid nanoparticles through Ugi‐mediated polymerization‐induced self‐assembly (U‐PISA), combining aqueous Ugi four‐component polycondensation with PEG‐based stabilization. Polypeptoid nano‐objects were produced directly from amino acid‐derived building blocks via a one‐pot process under mild, open‐air conditions. The effects of PEG molar mass and feed composition on particle formation and morphology were systematically examined, providing access to tunable spherical and worm‐like nanostructures. The in situ formation of amphiphilic PEG‐polypeptoid species suggests that nanoparticle formation proceeds through a PISA‐like mechanism. However, the step‐growth nature of the Ugi polycondensation implies that both PEG‐functionalized and unconjugated polypeptoid chains may participate in nanoparticle assembly and stabilization. These results demonstrate that multicomponent polymerizations, such as Ugi polymerization, can be integrated into a PISA strategy, offering a simple route to polypeptoid nanoparticles with tunable morphologies and potential biomedical applications.

## Graphical abstract

Ugi‐mediated polymerization‐induced self‐assembly (U‐PISA) enables the one‐pot synthesis of PEG‐stabilized polypeptoid nanoparticles under mild aqueous conditions. By tuning PEG molar mass and feed composition, tunable spherical and worm‐like nano‐objects are obtained. This work demonstrates the potential of multicomponent step‐growth polymerizations as a versatile approach for generating functional polypeptoid nanoparticles.

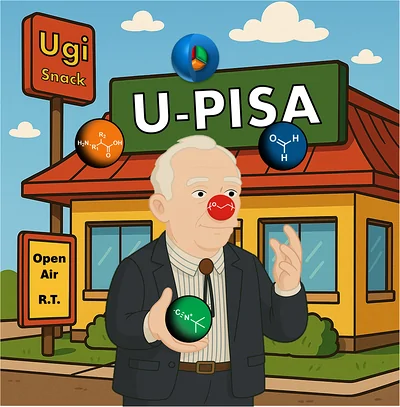

## 1 Introduction

Peptoids are peptidomimetic polymers that have emerged as an exceptionally significant class of synthetic materials with numerous biologically relevant properties, making them suitable for applications in many fields, especially in biomedicine [1]. They offer several advantages over natural polypeptides, including improved stability, resistance to enzymatic degradation, and the ability to tune their physical and chemical properties. There are different ways to synthesize polypeptoids, including ring‐opening polymerization (ROP) of α‐amino acid N‐carboxyanhydrides (NCAs) or derivatives, whose synthesis remains challenging, water‐sensitive, and involves multiple‐step reactions [2, 3]. Recently, multicomponent reactions (MCRs) have been introduced as new tools to synthesize polymers by step‐growth polymerization [4, 5]. MCRs such as the Passerini [6, 7, 8], Ugi [9, 10], and Biginelli [11] reactions are characterized by the association of three or more reactants in one pot to form single products under mild conditions. Lately, Ugi polycondensation has been described in the literature as a versatile route to produce polypeptoids [12]. The Ugi reaction forms a peptide‐like structure by combining an amine, a carbonyl compound (usually an aldehyde or ketone), an isocyanide, and a carboxylic acid. This reaction typically results in the formation of α‐amido‐amide motifs, making it useful in the synthesis of peptidomimetics and complex molecules [13]. In this way, Debuigne and coworkers developed a straightforward aqueous synthesis of alternating peptide‐peptoid stimuli‐responsive copolymers via a Ugi‐4 center‐3 component polymerization (Ugi‐4CP) applied to dipeptides [14]. Functional peptide‐peptoid copolymers as well as block copolymers were produced accordingly in a single step using glycyl‐glycine, glycyl‐alanine, or L‐alanyl‐glycine. Zhang et al. demonstrated that Ugi reaction of natural amino acids such as Nα‐Boc‐L‐ornithine, Nα‐Boc‐L‐lysine, or L‐glutamic acid methyl ester leads to structurally diverse polypeptoids under mild conditions in methanol [15]. These polypeptoids exhibit good biocompatibility and antibacterial activity, and can be used as a drug delivery system. Lately, Debuigne et al. developed a straightforward one‐step synthesis of polypeptoid analogues via Ugi‐4CP of amino acid derivatives in water [16]. Diverse polypeptoids with pH‐responsiveness and thermo‐responsiveness with low cytotoxicity were obtained.

Peptoids are often formulated as nanoparticles for applications in biomedical fields, including targeted drug delivery and antimicrobial treatments [17, 18, 19]. For numerous applications including biomedicine, formulation of nanoparticles from polypeptoids generally requires a nanoprecipitation involving toxic organic solvents and relatively dilute conditions (<1% by mass) or a tedious solvent emulsion‐evaporation strategy [20]. Over the past decades, polymerization‐induced self‐assembly (PISA) has emerged as a straightforward approach for preparing polymer particles and assemblies with controlled sizes and morphologies, under dispersion or emulsion polymerization conditions, without the need for external organic solvent [21]. A wide range of nano‐objects, including spherical micelles, worm‐like micelles and vesicles, can be efficiently prepared by in situ chain‐extension of the solvophilic macro‐chain with a solvophobic block in either polar or non‐polar solvents at high solids content (up to 50% w/w). Many chain‐growth polymerization techniques have been applied to PISA, such as Reversible Addition‐Fragmentation chain Transfer (RAFT), Atom Transfer Radical Polymerization (ATRP), Nitroxide‐Mediated Polymerization (NMP), Iodine transfer polymerization (ITP), Ring‐Opening Metathesis Polymerization (ROMP), and more recently ROP [22, 23, 24]. The latter method notably allowed to obtain polypeptide particles from N‐carboxyanhydride (NCA) monomers [23]. Lately, our group reported the RAFT PISA of methacrylic monomers initiated from a poly(ε‐L‐lysine) macroRAFT agent preformed by Ugi‐4CP, leading to polypeptoid‐decorated nanoparticles [25].

Although PISA has been predominantly developed using controlled chain‐growth polymerization techniques, it is not intrinsically restricted to such mechanisms [26]. Yet, genuine step‐growth‐based PISA strategies remain unreported, with only a limited number of related concepts explored, mainly through surfactant‐assisted emulsion polycondensation systems [27, 28]. This gap can be explained by several intrinsic features of step‐growth polymerization processes. In the particular case of polycondensation, water can shift the reaction equilibrium, reducing monomer conversion and limiting the molecular weight of the resulting polymers. Moreover, unlike chain‐growth polymerizations, where the growing block remains covalently attached to a hydrophilic macrochain, step‐growth polymerizations produce a mixture of chains, including free hydrophobic polymers that are not linked to the hydrophilic segment, precluding the exclusive formation of well‐defined amphiphilic diblock which assemble as in conventional PISA systems. In this context, novel step‐growth‐based polymerization approaches remain to be explored to determine whether such intrinsic limitations can be mitigated, and whether they can still lead to unique nano‐objects with tunable morphologies.

In this work, we investigate the potential of Ugi multicomponent polycondensation to evolve into a PISA process, here referred to as Ugi‐mediated PISA (U‐PISA), and to produce in a single step polypeptoid nano‐objects from amino acids in water (Scheme 1). The influence of the molar mass of the hydrophilic macrochain and of its stoichiometric ratio with the Ugi components is discussed in relation to the morphology of the resulting particles. Effort is also devoted to shedding light on the underlying mechanism in the present system, providing insights into the ability of Ugi multicomponent polymerization to couple polypeptoid synthesis and self‐assembly.

**SCHEME 1:**
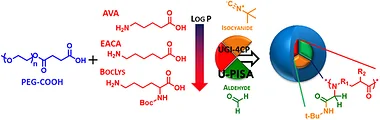
Preparation of stable particles using aqueous Ugi‐Mediated Polymerization‐Induced Self‐Assembly approach (U‐PISA) using Ugi‐4CP with PEG‐COOH and three different amino acids.

To investigate the concept of U‐PISA and verify its capacity to produce polypeptoid‐based particles in a single step in water, we carried out the Ugi‐4CP using tert‐butyloxycarbonyl‐lysine (BocLys), a water‐soluble modified natural amino acid, along with formaldehyde (CH_2_O) and tert‐butyl isocyanide (BuNC) in the presence of carboxylic acid‐terminated polyethylene glycol (PEG2k‐COOH) as a steric stabilizer. This PEG derivative was prepared by esterification of commercial hydroxy‐terminated PEG (PEG2k‐OH) with succinic anhydride (Figure S1). This approach is designed to generate the corresponding PEG‐polypeptoid copolymer in situ, which self‐assembles and stabilizes the formation of polypeptoid particles in water.

In practice, BocLys and CH_2_O were first mixed in water with PEG2k‐COOH. These molecules are soluble in water and generate a continuous liquid phase at the beginning of the synthesis. The weight fraction of the initial Ugi components to water has been set to 22% in all experiments. Several minutes later, by adding the last Ugi constituent, namely BuNC, a stable emulsion appeared quickly (Figure 1A, experiment P3 and Video S1). Control experiments showed that BuNC alone exhibits limited miscibility with water but does not generate turbidity or a stable dispersion, indicating that the observed self‐assembly cannot be attributed solely to the poor aqueous compatibility of BuNC. Instead, the formation of stable dispersion likely results from the combined presence of the Ugi components, which promotes the generation of hydrophobic intermediates during the early stages of the reaction. By comparison, the same reaction without PEG2k‐COOH (P1) or with unmodified poly(ethylene glycol) methyl ether with a hydroxyl end chain (PEG‐OH) (P2) mixed in the same way did not lead to a stable emulsion as depicted in Figure 1A. Instead, a white precipitate appeared in the vial in accordance with previous work [16]. To further characterize these experiments and deepen our understanding, we performed size exclusion chromatography (SEC) of the three samples. The macromolecular characteristics are presented in Table S1. Under all conditions, the formation of oligomers or polymers was observed (Figure 1A and Figure S2). Concerning the Ugi experiments without PEG (P1), a polymer with a number average molar mass (*M_n_*) of 1.8 kg/mol and a dispersity (*Đ*) of 1.3 was obtained through PEG calibration on SEC with DMF (+NH_4_BF_3_ 0.5 mM) as eluent. When using PEG‐OH (P2), no notable change can be observed after the polymerization in the SEC chromatogram (Figure S2). In the presence of PEG2k‐COOH (P3), however, we predominantly observe a single population with a narrow dispersity (*Đ* = 1.12). Although the apparent number‐average molar mass determined by SEC (*M_n_* = 1.9 kg/mol) is only slightly higher than that of the PEG2k‐COOH precursor (*M_n_* = 1.6 kg/mol; *Đ* = 1.05). Indeed, the incorporation of polypeptoid segments may induce chain compaction, leading to an underestimation of the actual molar mass increase. Nevertheless, the disappearance of the precursor peak and the presence of a single, shifted pop^2^ulation are consistent with the formation of PEG‐polypeptoid conjugates. The structure of P1 and P3 was analyzed by ^1^H nuclear magnetic resonance (NMR) spectroscopy in DMSO‐d_6_ to emphasize the formation of the desired polypeptoid and PEG‐polypeptoid. Spectra are presented in Figure 1C. P1 shows typical signals of the polypeptoid repeating unit, including peaks of the tert‐butyl groups from BuNC and BocLys precursors at 1.2 and 1.4 ppm, respectively. The α‐amido amide moiety generated by the Ugi‐4CP is also highlighted by the presence of amidic protons around 7 ppm and the protons b, d and e next to amino groups at around 3.4, 3.7 and 4.2 ppm (Figure 1C).

**FIGURE 1:**
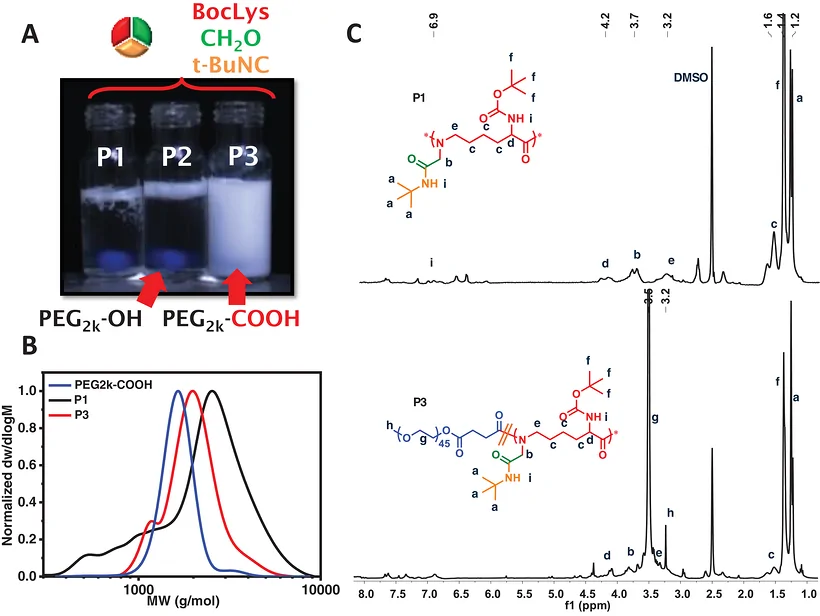
(A) Pictures of the Ugi‐4CP experiment vials with different components after a few minutes of polymerization (B) Size exclusion chromatograms in DMF of P1 and P3 and control PEG2k‐COOH (C) ^1^H NMR spectra of P1 and P3 in DMSO‐d_6_.

In the case of P3, PEG carboxylic acid proton peaks assigned to the terminal methyl ether at 3.2 ppm and the ethylene ether protons at 3.5–3.7 ppm can be observed. The ratio between PEG and lysine was further calculated by taking the ethylene ether integration belonging to the 182 protons of the PEG2k chain as reference and by integrating the two tert‐butyl peaks divided by 18, the number of protons belonging to the Ugi peptoid unit. The estimated PEG/Lysine ratios after Ugi polycondensation for the PEG‐polypeptoid polymers obtained are presented in Table S1. In the case of P3, this ratio is around 1:5.1, meaning that there is one PEG chain for 5.1 lysine units. Additional details regarding the NMR ratios are included in Table S1. This successful demonstration of the U‐PISA process highlighted the need for further studies to better understand and broaden the scope of this approach.

Nanoparticles (NPs) and stable colloid formation strongly depend on the hydrophilic/hydrophobic balance that eventually builds the NPs' core and the outer shell. The choice of PEG‐COOH/amino acid combination thus becomes crucial for the formation and even the morphology of NPs obtained during the self‐assembly. To assess the influence of the hydrophilic segment on the NPs formation and shape, a longer PEG (PEG5k‐COOH) was prepared and considered in the U‐PISA process. SEC analysis of the functionalized PEG5k‐COOH (Figure S1) revealed a minor shoulder corresponding to a small fraction of higher‐molecular‐weight species, most likely arising from PEG dimerization during the functionalization step. The resulting dimerized PEG is expected to behave as a spectator species during the subsequent Ugi polycondensation due to its reduced availability of carboxylic acid end groups and therefore should have only a minor impact on the overall polymerization and nanoparticle formation. Two new samples were prepared, P4 and P5, using 1:9 and 1:19 PEG5k‐COOH/BocLys ratios, respectively, and compared with previously described P3 with a 1:9 PEG2k‐COOH/BocLys ratio. Both P4 and P5 samples resulted in the formation of nanoparticles, as evidenced by the onset of turbidity within the polymerization solution. To evaluate the assemblies that arose from the U‐PISA process, the particles were characterized by Dynamic Light Scattering (DLS), Small‐Angle Neutron Scattering (SANS), and Transmission Electron Microscopy (TEM). These data can be seen in Figure 2; 1H NMR and SEC data are presented in Figure S3. TEM images show that all combinations of PEG/BocLys resulted in the formation of disperse spherical particles around 300 nm (Figure 2A and Figure S4). In the case of P5, atypical hollow spheres can be observed when the ratio of [PEG]:[AA] was reduced to 1:19 instead of 1:9. The construction of a histogram via inspection and counting of particles in the TEM images and comparison with DLS is in correlation with a moderately polydisperse distribution (Figure 2B). DLS measurements (Figure 2C) show hydrodynamic diameter (*D_h_*) values from 264 nm (P4) to 790 nm (P5), with corresponding polydispersity (PD) from 0.24 to 0.20. Morphology and size of NPs formed in the U‐PISA process thus strongly depend on the PEG length and its ratio to BocLys, which influence the hydrophilic/hydrophobic balance during self‐assembly. In addition, although hydrophobic interactions are considered the primary driving force, hydrogen‐bonding interactions between the amide groups of the polypeptoid backbone may also contribute to the cohesion and stabilization of the resulting nano‐objects. Longer PEG chains (PEG5k‐COOH in P4 (1:9 ratio) and P5 (1:19 ratio) lead to larger hydrodynamic diameters (264 nm for P4, 790 nm for P5) and distinct structures (i.e., hollow spheres).

**FIGURE 2:**
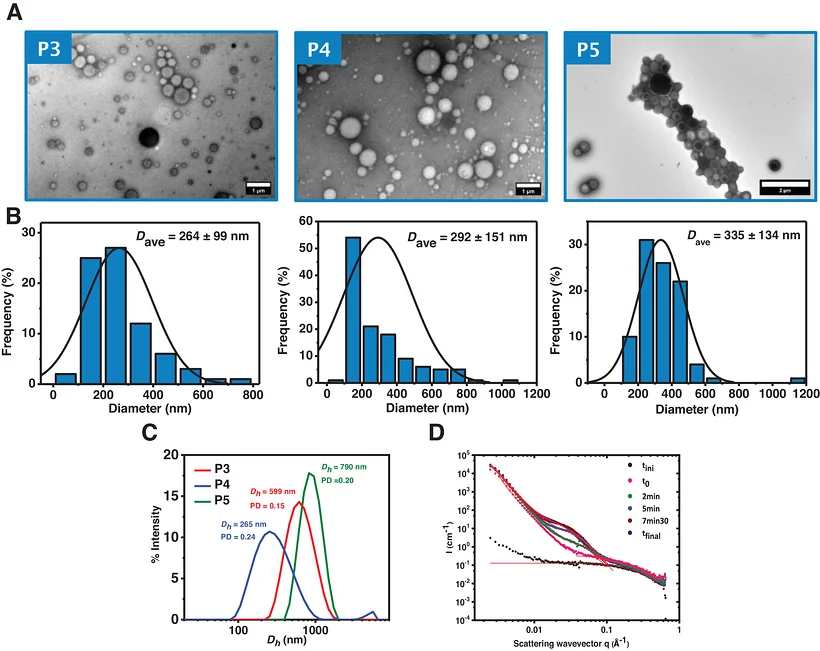
(A) Representative dry‐state TEM images of particles P3, P4, and P5, stained with 1 wt.% uranyl acetate, and (B) histograms of particles’ size distribution measured from particle analysis from TEM images. In each case, at least 100 particles were analyzed. (C) Hydrodynamic diameter distribution measured by DLS in MQ water. (D) Small‐angle neutron scattering (SANS) kinetics of P3 with fit lines in red.

The ^1^H NMR spectra of P4 and P5 closely resemble that of P3, indicating similar chemical environments (Figure S3A). In terms of molecular weights assessed by SEC, two distinct populations are observed for P4 (Figure S3B). One has a lower *M_n_* of 1.4 kg/mol, smaller than that of P1, and the other shows a *M_n_* of 4.7 kg/mol, slightly higher than PEG5k‐COOH alone. A similar trend is observed for P5, although the proportion of the lower molecular weight fraction is reduced. The presence of the higher molar mass population indicates that some of the growing Ugi‐derived polymers are covalently attached to PEG‐COOH, while the low molar mass peak corresponds to the formation of free polypeptoid oligomers. This observation is consistent with the characteristics of step‐growth polymerization, where, unlike chain‐growth polymerization, all monomers are equally reactive and can contribute to polymer formation.

SANS kinetics were conducted to monitor the formation of nanoparticles (NPs) for P3 in D2O. Prior to the initiation of the reaction, the scattering profile, corresponding solely to PEG‐COOH in solution, was well described by a Gaussian chain model, consistent with a low‐molar‐mass polymer exhibiting a radius of approximately 8 Å. Upon addition of the Ugi components, a pronounced q^−^^4^ slope emerged, characteristic of a sharp interface between scattering objects and the solvent, indicating the formation of emulsion droplets. This region of the curve was fitted using a Porod model, yielding an estimated average droplet radius of around 900 Å. While approximate, this large value suggests the presence of sizable emulsion droplets. At high *q* values, the polymer signal remains visible, while an increase in scattering intensity in the intermediate *q* range reveals the formation and growth of small spherical particles as the reaction proceeds. These evolving nanostructures were successfully modeled using a sphere model (details provided in Table S2), illustrating the dynamic self‐assembly process occurring during the reaction.

In summary, the length and ratio of the hydrophilic segment in the U‐PISA process play a decisive role in determining the size, morphology, and internal architecture of the nanoparticles. Longer PEG chains and higher PEG:BocLys ratios result in larger particles, distinct structures such as hollow spheres, and evolving assemblies during the reaction. These observations highlight the importance of carefully selecting and tuning PEG chains to achieve desired nanoparticle properties and functionalities.

In pharmaceutical sciences, hydrophilicity and hydrophobicity are commonly assessed using the partition coefficient (P), which describes the distribution of a compound between octanol and water. The logarithmic form, log P, indicates the compound's lipophilicity or hydrophobicity, with higher values corresponding to greater hydrophobicity. In a previous study, the log P parameter proved to be a useful predictor for identifying and evaluating new monomers for self‐assembly studies [29, 30]. However, monomer log P values alone cannot directly describe the effective hydrophilic/hydrophobic balance of the resulting polymers, which also depends on the repeat‐unit structure, the PEG block length, the polymer composition, and the overall amphiphilic architecture. In the present work, the amino acid log P values are therefore used only as qualitative descriptors of side‐chain hydrophobicity to guide monomer selection. Thus, to further investigate the influence of amino acid components on the U‐PISA process, we selected two new amino acids with lower log P values than BocLys: 5‐aminovaleric acid (AVA) and 6‐aminocaproic acid (EACA). The log P values of the selected amino acids are −0.4 for AVA, 0.02 for EACA, and 0.24 for BocLys. The new PEG‐polypeptoid systems synthesized from these amino acids exhibit distinct aspects compared to those previously observed with BocLys, including differences in their appearance in aqueous solution. Eight new polymers were synthesized (P6‐P13, Table S1). P6 and P7 used AVA and EACA components without PEG stabilizers and resulted in precipitation after a few minutes, as observed previously with P1. SEC and ^1^H NMR data can be seen in Figure S5. In samples P8, P9, and P10, AVA was combined with PEG2k‐COOH in a 1:9 ratio and with PEG5k‐COOH in 1:9 and 1:19 ratios, respectively. All of these led to colloidal suspensions as expected for the U‐PISA process. For samples P11, P12, and P13, EACA was employed in the same manner as AVA, using identical PEG types and ratios. Details of each polymer system can be found in Table S1. All ^1^H NMR data are consistent with polypeptoid formation (Figures S6 and S7).

Experiments using PEG2k‐COOH with AVA (P8) or EACA (P11) yielded nanoparticles with morphologies distinct from those previously obtained with BocLys. TEM analysis of P8 revealed very small nanoparticles, typically below 50 nm in diameter, while P11 showed the formation of worm‐like structures (Figure 3 and Figure S10). These observations were corroborated by DLS measurements, which confirmed the presence of distinct size distributions, and by SEC, which showed a molecular weight shift toward higher masses. These differences in nanoparticle shapes can be rationalized by considering the lower hydrophobicity (lower log P) of AVA and EACA, which reduces the overall hydrophobicity compared to BocLys of the growing block and consequently modifies the packing parameter during self‐assembly. In general, a less hydrophobic core‐forming block tends to decrease the packing parameter, favoring the formation of smaller or higher‐curvature morphologies such as spherical micelles or worms, rather than larger vesicles or aggregated structures. This relationship between monomer hydrophobicity, packing parameters, and resulting nanoparticle morphology is well documented in the literature and highlights the importance of monomer selection in controlling the outcome of the U‐PISA process [29].

**FIGURE 3:**
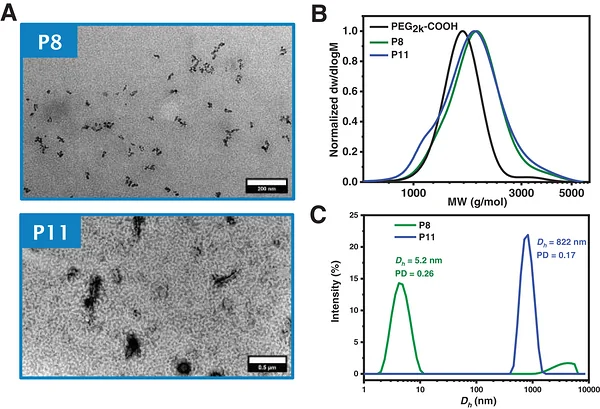
(A) Representative dry‐state TEM images of purified particles P8, P11, stained with 1 wt.% uranyl acetate; (B) Size exclusion chromatograms in DMF of P8 and P11 and control PEG2k‐COOH; (C) DLS.

For the other amino acid formulations using PEG5k‐COOH (P9, P10, P12, and P13), only spherical nanoparticles were observed by TEM (Figure S8), with sizes ranging from 87 nm for P9 up to 383 nm for P13. The ^1^H NMR spectra (Figures S6 and S7) appear consistent across samples, showing similar polypeptoid signatures, indicating successful incorporation of the amino acid units. In SEC (Figure S9), two distinct populations are again observed, as previously noted with BocLys‐based systems, suggesting partial attachment of the growing polymer chains to the PEG, alongside free oligomeric species.

These results highlight that both the amino acid‐to‐PEG ratio and the PEG chain length significantly influence the size and morphology of the resulting nanoparticles. While shorter PEG chains and more hydrophilic amino acids promote the formation of smaller or higher‐curvature structures (e.g., micelles or worms), increasing the PEG size or altering the stoichiometry by increasing the PEG‐to‐amino acid ratio can lead to larger spherical particles.

To summarize, we investigated Ugi‐Mediated Polymerization‐Induced Self‐Assembly (U‐PISA) as an approach to extend the PISA concept beyond conventional controlled chain‐growth polymerization methods. By combining aqueous Ugi multicomponent polycondensation with PEG‐COOH stabilization, we demonstrated the formation of stable polypeptoid nano‐objects, including spherical and worm‐like morphologies, directly from amino acid‐derived building blocks under mild and open‐air conditions.

From a mechanistic perspective, the self‐assembly process appears to share key features with conventional PISA systems, where the progressive formation of increasingly hydrophobic segments induces phase separation and drives nanoparticle formation. The formation of PEG‐containing polypeptoid structures, supported by SEC analysis and the influence of PEG molar mass and feed composition on particle morphology, suggests that covalently linked PEG‐polypeptoid species play a role in the stabilization of the resulting nano‐objects. However, unlike classical chain‐growth PISA processes, the step‐growth nature of the Ugi polymerization introduces additional complexity, as polymer chains can form both through PEG‐functionalized species and through reactions between small‐molecule precursors, leading to the possible presence of unconjugated polypeptoid chains. These species may nevertheless participate in the self‐assembly process by being incorporated into PEG‐stabilized aggregates, highlighting the distinct features of a step‐growth PISA mechanism.

The precise contribution of PEG‐conjugated vs. free polypeptoid species remains challenging to quantify and will require dedicated mechanistic investigations, including detailed compositional analysis of isolated assemblies and monitoring of species evolution during polymerization.

This complexity notwithstanding, the present study demonstrates that the Ugi‐4CP polymerization can be integrated into a PISA framework to generate stable polypeptoid nano‐objects. The versatility of the Ugi‐4CP, which allows independent variation of the aldehyde, amine, carboxylic acid, and isocyanide components, provides a powerful platform for future tuning of polymer structure, hydrophobicity, and nanoparticle morphology. More broadly, this work expands the scope of PISA‐related processes toward multicomponent, step‐growth‐based methodologies, opening new avenues for the design of functional and bioinspired polymer nanoparticles.

## Author Contributions

The manuscript was written through contributions of all authors. All authors have given approval to the final version of the manuscript.

## Conflicts of Interest

The authors declare no conflicts of interest.

## Supporting information




**Supporting File 1**: marc70389‐sup‐0001‐SuppMat.docx.

**Supporting File 2**: marc70389‐sup‐0002‐MovieS1.docx.

## Data Availability

The data that support the findings of this study are available from the corresponding author upon reasonable request.
