# Supplementary material for: Exploring a Ugi‐Mediated Polymerization‐Induced Self‐Assembly Approach to Peptoid Particles
Source: Macromol Rapid Commun. 2026 Aug 28;47(19):e70389. doi: 10.1002/marc.70389 (PMC13639226; doi:10.1002/marc.70389)
Supplement: Supplementary file 1 [file MARC-47-e70389-s001.docx]

Exploring A Ugi-Mediated Polymerization-Induced Self-Assembly Approach to Peptoid Particles

Thi Phuong Thu Nguyen,[a,b] Maria Cristina Molaro,[c] Hao-Ning Tien,[a] Lei Lei,[a,d] Pierre Stiernet,[e] Clemence Le Coeur,[a,f] Federica Sodano,[c] Antoine Debuigne,*[e] and Benoit Couturaud*[a]

[a]	Dr. T. Nguyen, H.-N. Tien, Dr. L. Lei, Dr. C. Le Coeur, Dr B. Couturaud

Université Paris Est Créteil, Institut de Chimie et Matériaux Paris-Est, UMR 7182

2-8 rue H. Dunant, F-94320, Thiais,

E-mail: [benoit.couturaud@cnrs.fr](mailto:benoit.couturaud@cnrs.fr)

[b]	Dr. T. Nguyen

Sorbonne Université, UMR 7574,

Laboratoire de Chimie de la Matière Condensée de Paris,

75005 Paris, France

[c]	M. C. Molaro, Dr. Federica Sodano

Department of Pharmacy, School of Medicine

University of Naples Federico II Via Domenico Montesano, 49

80131 Naples Italy

[d]	Dr. L. Lei

School of Automotive Materials, Hubei University of Automotive Technology,

Shiyan 442002, People’s Republic of China

[e]	Dr P. Stiernet, Dr. A. Debuigne

Center for Education and Research on Macromolecules (CERM),

CESAM Research Unit University of Liege (ULiege),

Quartier Agora, 13 Allée du Six Août, Sart-Tilman, B-4000 Liège, Belgium

E-mail: adebuigne@uliege.be

[f]	Dr. C. Le Coeur

Laboratoire Léon Brillouin, CEA-CNRS (UMR-12),

CEA Saclay, Université Paris-Saclay,

91191, Gif-sur-Yvette Cedex, France

KEYWORDS. Ugi, Polycondensation, Polymerization Induced Self-Assembly, Nanoparticle, Synthetic methods.

**Experimental section**

**1. Materials**

All chemicals were used as obtained without any further purification unless otherwise stated. Chemical reagents were purchased from Sigma-Aldrich as follows: formaldehyde solution (37 wt.% in H_2_O, contains 10-15% Methanol as stabilizer), tert-Butyl isocyanide (98%), 5-Aminovaleric acid (97%), 6-Aminocaproic acid (≥99%), Nα-Boc-L-lysine (99%), Poly(ethylene glycol) methyl ether (average Mn= 5kDa and 2kDa), succinic anhydride (≥99%), triethylamine (≥99.5%), methanol (MeOH, ≥99.8%, Aldrich), diethyl ether (Et_2_O, ≥99.7%), dichloromethane (CH_2_Cl_2_, ≥99.8%), dimethylformamide (DMF, ≥99.9%).

**2. Instrumentation**

**2.1. Polymer characterization**

Molecular weight determination of the precursor copolymers was obtained using size exclusion chromatography (SEC) in DMF as the eluent. SEC analyses were performed on a Shimadzu instrument fitted with mixed-C columns and RI. Molecular weight distributions (Mw/Mn) were calculated using polyethylene glycol (PEG) standards. All ^1^H NMR spectra were recorded on a Bruker 400 MHz FT NMR spectrometer. Samples were prepared in deuterated chloroform (CDCl_3_) or dimethylsulfoxyde (DMSO-d_6_) and their signals referenced to residual non-deuterated signals of the solvent. The spectra were analyzed using MestReNova 12.0.4 (Mestrelab Research S.L.).

**2.2. Particle characterization**

*Transmission electron microscopy (TEM)*

Dry-state TEM imaging was performed on FEI TECNAI F20 microscope at an acceleration voltage of 200 kV. All aqueous samples were diluted with deionized water and then deposited onto formvar-coated copper grids. After roughly 90 s, excess sample was blotted from the grid and the grid was stained with an aqueous 1 wt.% uranyl acetate (UA) solution for 90 s, prior to blotting, drying and microscopic analysis. Image J was utilized for image processing.

*Dynamic Light Scattering (DLS)*

The intensity-average sphere equivalent diameter of diblock copolymer nano-objects was determined at 25 °C by DLS using a Malvern Zetasizer Nano S instrument via the Stokes−Einstein equation, which assumes perfectly monodisperse, non-interacting spheres. All measurements were prepared at 0.1 wt.% in water. Z-average hydrodynamic diameters (DZ) were calculated from 3 repeat measurements.

*Small-Angle-Neutron Scattering (SANS)*

SANS data were measured on D22 [DOI:10.5291/ILL-DATA.9-10-1846] (url: <https://doi.ill.fr/10.5291/ILL-DATA.9-10-1846>) at the Institut Laue-Langevin – The European Neutron Source, Grenoble, France. The front detector was positioned at 1.4 m from the sample at an angle of 20°, the rear detector was at 17.6 m, collimated beam, source aperture of 40×55 mm² and a sample aperture of 7×10 (h×v) mm². A wavelength (λ) of 6.0 Å (relative full width at half maximum 10 %) was selected, allowing to cover a continuous q-range of 0.0025–0.65 Å^-1^ where q is the magnitude of the wavevector (q=4πsin(θ)/λ, 2θ being the scattering angle). Samples were kept in quartz cuvettes (type 100-QS, Hellma GmbH, Müllheim, Germany) of 1- or 2-mm pathway, on a thermalized rack with recorded temperature. Data were reduced with the program Grasp V.10.25f [DOI:10.1107/S1600576723007379] normalizing with monitor, subtracting the contribution from the empty cell, taking into account noise from the measurement with a sintered B_4_C piece at the sample position, and using for transmission the intensity transmitted by a semi-transparent beam stop. Parallax from the detector and from the sample attenuation were corrected for, and a flat field was used. Absolute scale was obtained from the measurement of the direct beam with a calibrated attenuator (flux of 1.64 MHz). Contributions from the solvent and incoherent scattering were then subtracted. The fitting software used was SasView 5.0.4. The polymers were fitted using a Gaussian chain model, whereas a polydisperse sphere model was applied during the reaction in the mid-q range.

For sphere without interactions, the scattering intensity is given by:

$I_{spheres}\left( q,r_{s} \right)=\frac{I_{0}}{V}[{V (\rho_{s}-\rho_{D20})]}^{2}P_{s}(q,r_{s})$	(1)

Where $\Phi$ is the volume fraction of polymeric structures, r_s_ and r_D2O_ are the respective neutron scattering length density of the polymer and of D_2_O, V is the volume of the polymer sphere; $P_{s}\left( q,r_{s} \right)$is the normalized sphere form factor. Fits were performed with the help of the SasView software (https://www.sasview.org). Polydisperse sphere model describes spheres with a log-normal distribution of the radius particle r_s_ (Eqs. 1 and 2) with:

$P_{s}\left( q,r_{s} \right)=9\left( \frac{[sin(qr_{s})-qr_{s}cos(qr_{s})]}{{(qr_{s})}^{3}} \right)^{2}$	(2)

Where r_s_ is the radius of the sphere.

**3. Synthesis procedures**

*PEG carboxylic acid derivatives synthesis*

*Typical synthesis of PEG2k/5k succinic acids (PEG2k-COOH/PEG5k-COOH)*

MeO-PEG_45_-OH (2k Da) (5.0 g, 2.5 × 10^-3^ mol) was dissolved in DMF (25 mL) in a 100 mL round bottom flask. Succinic anhydride (500.4 mg, 5.0 × 10^-3^ mol) and TEA (697 μL, 5.0 × 10^-3^ mol) was added to the flask subsequently. The reaction mixture appeared pinkish-orange and transparent after the complete dissolution of solid and was stirred and heated at 45 °C overnight. The reaction mixture was then cooled down to room temperature and transferred to a 1L Erlenmeyer flask. Cold diethyl ether (400 mL) was added to the crude solution. The Erlenmeyer flask containing the reaction mixture was placed in a fridge at -20 °C for 1 h. The white precipitate was recovered from the bulk solvent by filtration the usage of a Büchner funnel and a grade 3 qualitative filter paper. The precipitate was dissolved again in MeOH (10 mL) and drop by drop added to a 1 L Erlenmeyer flask prefilled with cold diethyl ether (400 mL) by a glass syringe with a speed of 1 drop/s. The mixture was placed again in a fridge -20 °C for 1 h and then the white precipitate was recovered again from the bulk solvent by filtration. The purified product was dried overnight under vacuum. A white solid (4.148 g, 2.1 × 10^-3^ mol), MeO-PEG_45_-O(CO)C_2_H_4_COOH, named PEG2k-COOH, was obtained. ^1^H NMR spectroscopy was carried out to verify the purity of the functionalized polymer.

^1^H NMR (400 MHz, CDCl_3_) δ 4.23 (d, J = 3.2 Hz, 2H), 3.79 (d, J = 4.4 Hz, 2H), 3.63 (d, J = 9.2 Hz, 182H), 3.36 (d, J = 0.9 Hz, 3H), 2.61 (dd, J = 8.9, 4.5 Hz, 4H).

Same protocol has been conducted for the synthesis of MeO-PEG_113_-O(CO)C_2_H_4_COOH, named PEG5k-COOH, with MeO-PEG_113_-OH (5 kDa) (3.0 g, 6.0 × 10^-4^ mol), DMF (50 mL), succinic anhydride (141.71 mg, 1.2 × 10^-3^ mol) and TEA (167 μL, 1.2 × 10^-3^ mol). A white solid (2.593 g, 5.2 × 10^-4^ mol) was obtained. ^1^H NMR spectroscopy was carried out to verify the purity of the functionalized polymer.

^1^H NMR (400 MHz, CDCl_3_) δ 4.24 (s, 2H), 3.80 (t, J = 4.4 Hz, 2H), 3.64 (d, J = 12.6 Hz, 454H), 3.37 (s, 3H), 2.62 (dd, J = 10.1, 4.4 Hz, 4H).

**Figure S1.** ^1^H NMR spectrum in CDCl_3_ (A) of PEG2k-COOH and (B) PEG5k-COOH and (C) SEC chromatogram in DMF of PEG2k-COOH and PEG5k-COOH and corresponding unmodified PEG.

*General synthesis of PEG-COOH/polypeptoid polymers*

PEG-COOH (2k and 5kDa) was dissolved in distilled water (PEG+Amino Acid = 1 equivalent) in a 1 mL glass vial. A sonicator was used to facilitate the solid dissolution. Amino acid (*AVA*, *EACA* or *BocLys*) was added to the glass vial. Formaldehyde (1.1 equivalent) was added to the mixture subsequently. After another sonication of 5 min and stirring, *t*-butyl isocyanide (1.3 equivalent) was added to the glass vial in a fumehood. The reaction mixture was then stirred at room temperature for 24 h.

The total amount of liquid of each essay varied according to the wt.% of main Ugi component, which is defined as the sum of weight of the amino acid (AA), formaldehyde (CH_2_O) and tert-butyl isocyanide (BuNC). In addition, the total amount of liquid was the sum of volume of the added distilled water, formaldehyde and TB-NC. In all of the essays conducted in this work, wt.% was maintained at approximately 22 wt.%, that is:

wt.% = (m_AA_+m_CH2O_+m_Bu-NC_) / (V_H2O_+V_CH2O_+V_Bu-NC_)*100

Therefore, V_H2O_ was the amount of distilled water required as a solvent.

For example, P3 has been synthesised as followed: PEG2k-COOH (120 mg, 60 µmoL, 0.1 eq) and BocLys (133 mg, 540 µmol, 0.9 eq) was dissolved in 870 µL of distilled water. Formaldehyde (53.5 µL, 660 µmoL, 1.1 eq) was added to the mixture subsequently. After another sonication of 5 min and stirring, t-butyl isocyanide (47.7 µL, 780 µmoL, 1.3 eq) was added.

*Purification protocol for NMR and SEC*

Water was removed from the crude mixture by freeze-drying. The dried crude mixture was then dissolved in a minimum amount of MeOH (less than 0.5 mL), sonicated for 5 min. The solution was drop-by-drop added to a 20 mL glass vial prefilled with cold Et_2_O (~18 mL) by a glass Pasteur pipette. The glass vial was placed in a fridge at -20 °C for 1 h to 24 h until the observation of white precipitation and a clear, transparent supernatant solution. The supernatant Et_2_O was removed by a glass Pasteur pipette, in order not to destabilize the precipitation. The purification procedure with Et_2_O was repeated three times. Eventually, the white precipitate was dried under vacuum overnight.

**Table S1.** Polymers obtained by U-PISA

| Entry | PEG | AA | PEG:AA | Peak 1^[a]^ | | Peak 2 | | PEG:AA after U-PISA^[b]^ | Theoritical peptoid average molar mass (kDa) |
| --- | --- | --- | --- | --- | --- | --- | --- | --- | --- |
|  |  |  |  | M_n_ (kDa) | *Đ* | M_n_ (kDa) | *Đ* |  |  |
|  | PEG2kCOOH |  |  | 1.6 | 1.05 |  |  | - |  |
|  | PEG5kCOOH |  |  | 4.3 | 1.12 |  |  | - |  |
| P1 | - | BocLys | - | 1.8 | 1.3 | - | - | - |  |
| P2 | PEG2k-OH | BocLys | 1:9 | 1.5 | 1.05 | - | - | - |  |
| P3 | PEG2k-COOH | BocLys | 1:9 | 1.9 | 1.12 |  |  | 1:5.1 | 1.7 |
| P4 | PEG5k-COOH | BocLys | 1:19 | 4.7 | 1.11 | 1.4 | 1.05 | 1:7.8 | 2.7 |
| P5 | PEG5k-COOH | BocLys | 1:9 | 4.7 | 1.04 | 1.4 | 1.05 | 1 :8.1 | 2.8 |
| P6 | - | AVA | - | 0.8 | 1.40 | - | - | - |  |
| P7 | - | EACA | - | 1.0 | 1.30 | - | - | - |  |
| P8 | PEG2k-COOH | AVA | 1:9 | 1.9 | 1.08 |  |  | 1:3.5 | 0.7 |
| P9 | PEG5k-COOH | AVA | 1:19 | 4.7 | 1.04 | 1.4 | 1.17 | 1:2.7 | 0.6 |
| P10 | PEG5k-COOH | AVA | 1:9 | 4.5 | 1.10 | 1.1 | 1.20 | 1:4.9 | 1.0 |
| P11 | PEG2k-COOH | EACA | 1:9 | 1.9 | 1.11 |  |  | 1:7.2 | 1.6 |
| P12 | PEG5k-COOH | EACA | 1:19 | 4.8 | 1.05 | 1.3 | 1.04 | 1:5.0 | 1.1 |
| P13 | PEG5k-COOH | EACA | 1:9 | 4.5 | 1.10 | 0.96 | 1.36 | 1:12.1 | 2.7 |

[a] Number average molar mass (M_n_) and dispersity (*Đ*) determined by SEC using DMF as eluant and Refractive Index detection. [b] Ratio determined by ^1^H NMR after polymerization and purification

**Table S2.** Sphere radius obtained by SANS.

| **Time** | **R_g_ of polymer**  **(in Å)** | **Radius of sphere**  **(in Å)** | |
| --- | --- | --- | --- |
|  |  |  |  |
| T_ini_ | 8 |  |  |
| T_o_ | 8 | 21 |  |
| 2 min | 8 | 50.2 |  |
| 5 min | 8 | 53.1 |  |
| 7min30s | 8 | 59.3 |  |
| T_final_ | 8 | 65 |  |
|  |  |  |  |

**Figure S2.** (A) ^1^H NMR spectrum in DMSO-d_6_ and (B) SEC chromatograms of P2 using DMF as eluant.

**Figure S3.** (A) ^1^H NMR spectra in DMSO-d_6_ of P4 and P5. (B) SEC chromatograms of P4 and P5 using DMF as eluant.

**Figure S4.** Representative dry-state TEM images of particles P3, P4 and P5, stained with 1 wt% uranyl acetate.

**Figure S5.** (A) ^1^H NMR spectra in DMSO-d_6_ and (B) SEC chromatograms of P6 and P7 using DMF as eluant.

**Figure S6.** ^1^H NMR spectra in DMSO-d_6_ of U-PISA polymers made from AVA.

**Figure S7.** ^1^H NMR spectra in DMSO-d_6_ of U-PISA polymers made from EACA.

**Figure S8.** Representative dry-state TEM images of particles P9, P10, P12 and P13, stained with 1 wt% uranyl acetate.

**Figure S9.** SEC chromatograms of P9, P10, P12, P13 using DMF as eluant.

**Figure S10.** Representative dry-state TEM images of purified particles P11, stained with 1 wt% uranyl acetate.
